# CircRNA NRIP1 promotes papillary thyroid carcinoma progression by sponging mir-195-5p and modulating the P38 MAPK and JAK/STAT pathways

**DOI:** 10.1186/s13000-021-01153-9

**Published:** 2021-10-25

**Authors:** Chuang Li, Lijuan Zhu, Lijun Fu, Mingli Han, Ya Li, Zhaozhong Meng, Xinguang Qiu

**Affiliations:** 1 Department of Thyroid Surgery, The First Affiliated Hospital of Zhengzhou University, Νo. 1 Jianshe East Road, 450052 Zhengzhou, China; 2 Department of Thyroid and Neck, Henan Cancer Hospital, The Affiliated Cancer Hospital of Zhengzhou University, 450000 Zhengzhou, China; 3 Department of Ophthalmology, The First Affiliated Hospital of Zhengzhou University, 450052 Zhengzhou, China; 4 Department of Breast Surgery, The First Affiliated Hospital of Zhengzhou University, 450052 Zhengzhou, China; 5 Institute for Respiratory Diseases, The First Affiliated Hospital of Henan University of Chinese Medicine, Henan 450000 Zhengzhou, China

**Keywords:** Papillary thyroid carcinoma, CircRNA NRIP1, miR-195-5p, P38 MAPK pathway, JAK/STAT pathway

## Abstract

**Background:**

Circular RNAs (circRNAs) have become a hot topic in the area of tumor biology due to its closed structure and the post-transcriptional regulatory effect. This study aims to clarify the roles of circRNA nuclear receptor-interacting protein 1 (NRIP1; circNRIP1) and the possible mechanisms in papillary thyroid carcinoma (PTC).

**Methods:**

The real-time PCR was used to detect the expression level of CircRNA NRIP1 in PTC specimens and cell lines. The effects of CircRNA NRIP1 and miR-195-5p on the PTC cell functions were detected by MTT, transwell, and flow cytometry assays. Dual-luciferase reporter assays and pull down assays were used to verify the association between circRNA NRIP1 and miR-195-5p. The murine xenograft models were constructed to detect the roles of CircRNA NRIP1 and miR-195-5p. Western blot was applied to detect the effects of CircRNA NRIP1 and miR-195-5p on the P38 MAPK and JAK/STAT singling pathways.

**Results:**

CircRNA NRIP1 was over-expressed in PTC tissues and cells and the high levels of CircRNA NRIP1 were correlated with advanced PTC stage. Depletion of CircRNA NRIP1 inhibited PTC cell proliferation, invasion, while accelerated apoptosis. miR-195-5p upregulation repressed proliferation and invasion capabilities, and accelerated apoptosis of PTC cell lines and restraining the growth of tumor xenografts, while the functions were reversed following CircRNA NRIP1 overexpression in PTC cells and tumor xenografts. Besides, the protein levels of p-p38, p-JAK2 and p-STAT1 were remarkably down-regulated in miR-195-5p overexpressed PTC cells and tumor xenografts, whereas CircRNA NRIP1 up-regulation overturned the impacts.

**Conclusions:**

In conclusion, CircRNA NRIP1 promoted PTC progression by accelerating PTC cells proliferation, invasion and tumor growth, while impeding apoptosis by way of sponging miR-195-5p and regulating the P38 MAPK and JAK/STAT pathways.

**Supplementary Information:**

The online version contains supplementary material available at 10.1186/s13000-021-01153-9.

## Background

Thyroid cancer (TC) is one out of a multitude tumor types in the endocrine system, for which thyroid cancer incidence rate is increasing year by year [1]. According to its histological characteristics, TC can be classified as four forms, namely papillary thyroid carcinoma (PTC), follicular thyroid carcinoma (FTC), anaplastic thyroid carcinoma (ATC) and Medullary thyroid carcinoma (MTC), in which PTC occupies a large proportion of all and happened mostly in countries with iodine-adequate or iodine-excess diet [2]. PTC is a well-differentiated tumor, but it can slowly lead to invasiveness, metastasis and lethality for a long time [3]. Although the early prognosis is good, the 5-year survival probability of PTC patient in terminal period patients remains low [4]. Hence, there is imminent to explore the underlying molecular mechanisms of PTC progression and to investigate more accurate diagnosis and treatment strategies.

Circular RNAs (circRNAs) have a covalent closed loop which can protect it from exonuclease, are abundant, conservative and stable and are a type of non-coding RNA [5]. Some researchers have used microarray or sequencing technology to analyze the expression profiling of circRNA in cancer tissue specimens and corresponding adjacent organizations, and found that abnormal expression phenomena are common in tumors. CircRNA has many functions, the most prominent of which is miRNA sponge effect or as competitive endogenous RNA (ceRNA), which plays a role in regulating miRNA target genes and then affects the occurrence and progression of illness [6]. For example, CircRNA_100290 reduces the level of FZD4 through miR-516b and the Wnt/β-catenin signaling pathway to facilitate the advancement of colorectal tumor [7]. Circ-itch inhibits the proliferation, and invasion of triple negative breast cancer *in vitro* and *in vivo* by serving as a miR-214 and miR-17 sponge [8]. CircTADA2A accelerates the osteosarcoma development by acting as a miR-203 A-3P sponge and regulating CREB3 expression [9]. Due to the closed structure and the post-transcriptional regulatory effect, circRNA has become a new star in the field of tumor biology.

Studies by Dong et al. [10] on circRNA nuclear receptor-interacting protein 1 (CircRNA NRIP1) has been published in which they describe that CircRNA NRIP1 may work as a carcinogene to facilitate the development of renal carcinoma via sponging miR-505 by motivating the AMPK and PI3K/AKT/mTOR pathways. The research by Xie et al. [11] also has demonstrated that CircRNA NRIP1 is overexpressed in breast cancer tumor tissues and cells, which functions as a sponge of miR-653 to restrain its function and accelerate the tumor deterioration. Peng et al. have used Array star miRNA target prediction software to confirm the diverse expression levels of circRNAs and miRNAs in PTC tumors and discovered that CircRNA NRIP1 is remarkably highly expressed in PTC patients [12]. Yet, research in the specific roles of CircRNA NRIP1 and its diagnostic value in PTC remain poorly illuminated.

In this study, we searched the expression profile of CircRNA NRIP1 in PTC tumor tissues and corresponding non-tumorous tissues and explored the possible effects and mechanism of CircRNA NRIP1 on PTC cells *in vitro* and mice xenograft models *in vivo*. Our discoveries afford an actual and academic basics for exploring a novel available therapeutic for papillary thyroid carcinoma.

## Materials and methods

### Patients & specimens

Human tumor specimens and normal appearing tumor-adjacent thyroid tissue samples were collected from 50 cases of papillary thyroid carcinoma patients who were confirmed by pathological diagnosis at the First Affiliated Hospital of Zhengzhou University from Jan. 2017 to Aug. 2019. The pathological examination of papillary thyroid carcinoma was confirmed by two pathologists. All patients have signed the informed consents files. The present experiment was conducted in conformity to the ethical standards of the revised Declaration of Helsinki and with the permission of the Medical Ethical Committee of the First Affiliated Hospital of Zhengzhou University.

All tumor tissues were immediately placed in liquid nitrogen after being isolated. Real-time PCR method was applied to explore the expression levels of CircRNA NRIP1.

### Cell culture

The normal human thyroid follicular epithelial cell line Nthy-ori 3-1 cells, the human papillary thyroid carcinoma cell lines TPC1, B-CPAP, IHH-4 and the HER293T cells were all from Cell Bank of the Chinese Academy of Sciences (Wuhan, China) and K1 was offered by Be Na Culture Collection. Nthy-ori 3-1 and TPC1 cell lines were incubated in DMEM medium (Hyclone, USA) complemented with 15 % fetal bovine serum (FBS; GIBCO., Waltham, Massachusetts) and 1 % penicillin /streptomycin (GIBCO, Uxbridge, UK). B-CPAP, IHH-4 and K1 cell lines using the Roswell Park Memorial Institute-1640 medium (Hyclone, USA) comprised of 15 % FBS and 1 % penicillin /streptomycin. The HER293T cells were grown in DMEM supplemented with penicillin (100U/ml) /streptomycin (0.1 mg/ml) and 10 % FBS. All cells were kept in a humidified incubator (Thermo., Waltham, CA, USA) and constantly cultured at 37 °C in the humidity of 95 % air and 5 % CO_2_ atmosphere.

### Quantitative realtime polymerase chain reaction (qRT-PCR)

5 µg of total RNA was incubated with or without 3 U/µg RNase R (Epicentre Technologies, Madison, WI, USA) for 15 min at 37 °C. After treatment with RNase R, CircRNA NRIP1 levels were detected by qRT-PCR. Reverse transcriptional reaction was conducted with a PrimeScript™ RT reagent Kit (Promega, Madison, WI) or TaqMan MicroRNA Reverse Transcription Kit (Promega) according to the manufacturer’s manual. qRT-PCR reactions involved employing the Light Cycler-DNA Master SYBR Green qPCR mixture (Bio Rad, Hercules, CA) for miRNA and the Premix Ex Taq™ II (Bio Rad, Hercules, CA) for mRNA with a Biosystems 7500 fast Real-Time PCR System (Thermo, CA, USA). The qPCR amplification conditions included a 5 min denaturation at 95 °C, followed by 40 cycles of denaturation at 95 °C for 15 s, annealing at 60 °C for 30 s, and extension at 60 °C for 1 min. Fluorescent signals were normalized to *U6* and *GAPDH* for miRNA and circRNA, respectively. Then the 2^^−∆∆Ct^ method was applied to valuate and quantify the relative expression levels. The sequences of primers were shown in the supplementary Table 1.

### Dual-Luciferase reporter assay

The genome DNA of 293T cells was used as template for amplification of plasmids (Wild-type (WT) CircRNA NRIP1) contained the possible miR-195-5p binding sites and then plasmids CircRNA NRIP1-WT were used as template for amplification of mutant-type (MUT) and the primers used were showed in supplementary Table 1. And the plasmids were cloned into the pmir-GLO dual luciferase reporter vector (Promega, MD, USA) to construct (CircRNA NRIP1-WT and CircRNA NRIP1-MUT). For the luciferase assay, 5 × 10^5^ TPC1 and IHH-4 cells were seeded into 24-wells and co-transfected with CircRNA NRIP1-WT or CircRNA NRIP1-MUT and miR-195-5p or miR-NC using Lipofectamine 2000 (Invitrogen; Thermo., MD, USA). Following transfection 48 h, cells were treated with Luc-Pair™ Duo-Luciferase HS Assay Kit (Promega, Madison, WI, USA). The luciferase intensity in cells was analyzed using the utilized the Dual Luciferase® Reporter Assay System (Promega, MA, USA) in conformity to the standard protocols.

### RNA pull-down assay

TPC1 and IHH-4 cells were transfected with biotin-coupled miR-195-5p using lipo2000 (Invitrogen). Then, cells were harvested and lysed in lysis buffer. Streptavidin-conjugated magnetic beads were activated and blocked with blocking buffer for 3 h. And then, the beads were incubated with cell lysates overnight at 4 °C. The pull-down complexes were collected and measured by qRT-PCR.

### Plasmid construction and infection

All plasmids and oligonucleotides were synthesized including CircRNA NRIP1 overexpression lentiviral plasmid (NRIP1) or the corresponding negative controls (NC), short hairpin RNA against CircRNA NRIP1 (sh-CircRNA NRIP1) or a non-specific shRNA (sh-NC), miR-195-5p or miR-NC. sh-NRIP1, NRIP1 and miR-195-5p were cloned into pLenti-hU6-EF1-GFP-Puro, pLC5-ciR, pCDH-CMV-MCS-EF1-copGFP-T2A-Puro vectors, respectively. Then, they were transfected into 293T cells to package viruses expressing sh-NRIP1, CircRNA NRIP1 and miR-195-5p by calcium phosphate transfection reagent for further experiment. TPC1 and IHH-4 cells were infected with the above viruses.

### Cell proliferation assay

The infected TPC1 and IHH-4 cells (1 × 10^4^ cells per well) were collected, sowed with the 96-well plate in triplicate and cultivated for 0, 24, 48, 72 h, respectively. Each well was added 20 µl MTT reagent from MTT Cell Proliferation/Viability Assay Kit (Takara, Beijing, China) in accordance with the instruction. Another 4 h of incubation, then 150 µl DMSO were added into per well. Finally, the absorption rate at 490 nm was read by the microplate reader (Bio- Rad Laboratories, MA, USA).

### Apoptosis assay

The apoptotic rate of PTC cells was detected via an Annexin V-PE/7-AAD apoptosis detection kit (eBioscience, Carlsbad, MA, USA). Totally, the transfected TPC1 and IHH-4 cells (1 × 10^4^) were collected, washed and re-suspended after 48 h incubation, and then co-incubated with 5 µl Annexin V-PE and 5 µl 7-AAD in the dark for 20 min, following detected on the flow cytometer (FAC Scan, BD Biosciences, MA, USA). The apoptotic cell proportion was analyzed and calculated through EXPO32 ADC software (BD Biosciences).

### Cell invasion assay

Transfected TPC1 and IHH-4 cells (5 × 10^4^ cells per well) were resuspended with 300 µl serum-free medium and added to the top chamber of 24-well Transwell assay plates coated with Matrigel-coated membrane (8 mm pores, BD Biosciences, USA), and the bottom chambers was supplement with 500 µl of 10 % FBS medium. Followed by 24 h conventional cultivation, the non-invaded cells were scrubed using a wet cotton swab and the bottom cells were washed twice with PBS, immobilized with 4 % paraformaldehyde and dyed with 0.1 % crystal violet for 15 min, and again washed twice. Finally, we choose five randomly fields to count cells and digitally imaged using a microscope (Olympus, Corp, Japan).

### Animals and treatments

Female BALB/c nude mice (5 weeks old, 23.6±3 g; Henan Provincial Laboratory Animal Center, China) were randomly divided into four groups (N= 5/group): miR-195-5p, miR-NC, miR-195-5p + Lv-NC, miR-195-5p + CircRNA NRIP1. Then 300 µL of the transfected TPC1 cells with a density of (4 × 10^6^ cells per 1 mL PBS) was injected subcutaneously under the axilla of the mice. The feeding environment of the mice was of SPF grade referring to the NIH USA Guidance for the Care and Use of Laboratory Animals. Tumor volume was monitored every 5 days and computed via the formula: (length× width^2^)/2. Following 35 days inoculation, the animals were euthanized and xenografts were preserved at -80 °C for further analyses. Animal study was conducted in conformity to the Animal Protection Legislation and authorized by the Ethics committee of the institutional animal care and use at the Zhengzhou University.

### Immunohistochemistry staining assay

The tumor samples were fixed in 4 % paraformaldehyde, embedded into paraffin and then parceled in 3-µm sections. After deparaffinized, hydrated and blocked treatment (5 % BSA), the tissues were incubated with antibody against Ki67 (ab16667; Abcam) at 4 °C overnight, then added the secondary HRP-conjugated antibody (ab214880; Abcam) and incubated at room temperature for 60 min. DAB solution (DA1015; Solarbio, Beijing, China) was used to produce a dark brown reaction product. Eventually, counterstaining was conducted with hematoxylin reagent (Takara, Beijing, China) and the slides were visualized under a fluorescence microscope (Leica DMI 3000 B, Leica, Germany). Image J software was used for analyzing these images.

### Western blot analysis

The PTC tissues and cells lysates were collected using RIPA buffer (Invitrogen, shanghai, China) and protease inhibitor cocktail (#5871; Beverly, MA, USA). Protein concentrations were calculated using Bicinchoninic Acid Kit (Takara; Merck KGaA) referring to the manufacturer’s protocols. Immunoblotting procedures followed the previously published method [13]. Equal amounts (60 µg) of total tissues or cellular proteins were added into loading buffer, boiled for 5 min, and separated on 10 % SDS-PAGE electrophoresis. Subsequently, the proteins were metastasized to nitrocellulose (NC) membranes. Then we blocked the membranes with 5 % non-fat dry milk for 1 h at room temperature and hatched overnight with primary antibodies. Following further incubated with secondary antibody for 1 h, the immunoreactive protein strips were visualized by ECL kit (Thermo, CA, USA) on the Bio-Rad Chemical Doc XRS system (Bio-Rad). The primary antibodies were including anti-p-p38 (ab195049; 1:2000), anti-p38 (ab250612; 1:1000), anti‐p-JAK2 (ab32101; 1:1500), anti‐JAK2 (ab39636; 1:800), anti‐p-STAT1 (ab215820; 1:1000), anti‐STAT1 (ab155933; 1:1000) and anti‐β‐actin (ab179467; 1:5000) were purchased from Abcam.

### Statistical analysis

All experiments were repeated independently at least three times. Statistical analysis was implemented by utilizing SPSS 19.0. Student’s *t-*test or ANOVA was conducted in accordance to the actual conditions. Pearson’s correlation coefficient was applied to analyze the correlations between the expression of CircRNA NRIP1 and miR-195-5p in PTC patients. All experimental data were calculated as the mean ± SD. A *P* < 0.05 was considered statistically significance.

## Results

### CircRNA NRIP1 level is high in PTC tissues and cells

**Fig. 1:**
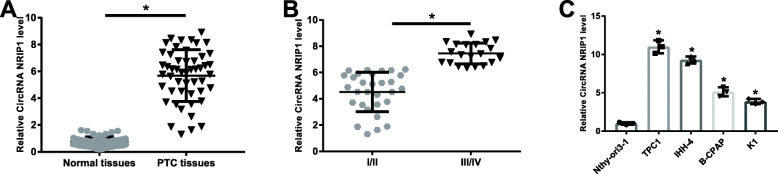
CircRNA NRIP1 levels were elevated in PTC tissues and cells. **A** The expression levels of CircRNA NRIP1 in PTC tissues ( *N* = 50) and corresponding normal tissues were determined using qRT-PCR. **B** The expression levels of CircRNA NRIP1 in stage I/II PTC patients and stage III/IV PTC patients via qRT-PCR. **C** The expression levels of CircRNA NRIP1 in PTC cell lines, including TPC1, B-CPAP, IHH-4 and K1 and the normal thyroid epithelial cell line Nthy-ori3-1 by qRT-PCR. *P < 0.05

CircRNA NRIP1 is reported to act as a tumorigenic CircRNA in renal carcinoma and esophageal squamous cell cancer [10, 14]. Figure 1A revealed that CircRNA NRIP1 was enhanced in PTC tumor tissues in comparison to the matched adjacent normal thyroid tissues. As shown in Fig. 1B, CircRNA NRIP1 was significantly elevated in the stage III/IV PTC patients. Moreover, the abundance of CircRNA NRIP1 was distinctly increased in various PTC cells, including TPC1, B-CPAP, IHH-4 and K1 cells, with TPC1 and IHH-4 cells expressing the highest level of CircRNA NRIP1, for which were used for the subsequent experiments (Fig. 1C). Therefore, we guessed that CircRNA NRIP1 probably was involved in PTC progression.

### CircRNA NRIP1 directly sponges to miR-195-5p

**Fig. 2:**
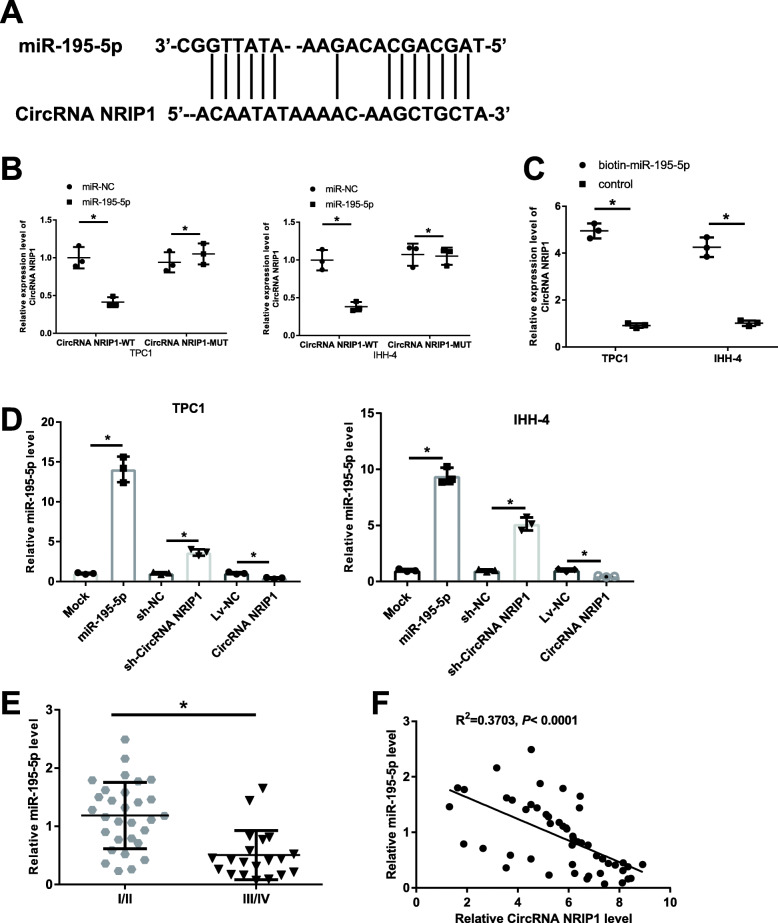
CircRNA NRIP1 directly sponges miR-195-5p and negatively regulated its expression, and miR-195-5p levels were reduced in stage III/IV PTC tissues and CircRNA NRIP1 was negatively correlated with miR-195-5p expression in PTC patients. **A** The predicted interaction sites of miR-195-5p and CircRNA NRIP1. **B** Analysis of the influence of CircRNA NRIP1  on miR-195-5p  expression using dual-luciferase reporter assay in TPC1 and IHH-4 cells. **C** Pull-down assay for analyzing the interaction relationship between labeled miR-195-5p and CircRNA NRIP1 in TPC1 and IHH-4 cells. **D** The expression levels of miR-195-5p in TPC1 and IHH-4 cells following the transfection of with CircRNA NRIP1, sh-NRIP1, miR-195-5p or its controls were detected through qRT-PCR. **E** The expression levels of miR-195-5p in stage I/II PTC patients and stage III/IV PTC patients via qRT-PCR. **F** Pearson’s correlation coefficient was applied to analyze the relationship between CircRNA NRIP1 and miR-195-5p expression in PTC patients. *P < 0.05

To examine the circRNA-miRNA interaction pathways, bioinformatics database Star Base 2.0 (http://starbase.sysu.edu.cn/starbase2/index.php) was applied to forecast the miRNAs viable binding sites in CircRNA NRIP1. The on-line prediction software predicted that CircRNA NRIP1 contained conserved binding sites with miR-195-5p (Fig. 2A). Following luciferase report assays revealed that miR-195-5p overexpression transfected in combination with CircRNA NRIP1-WT segment significantly reduced the fluorescence intensity in TPC1 and IHH-4 cells, however, there has not impact on the luciferase activity of CircRNA NRIP1-MUT (Fig. 2B). Results from pull-down assays showed that miR-195-5p significantly enriched by CircRNA NRIP1 in cells (Fig. 2C). The production of miR-195-5p showed a significant elevation in TPC1 and IHH-4 cells transfected with miR-195-5p in comparison to the control. Moreover, overexpression of CircRNA NRIP1 or knockdown of CircRNA NRIP1 could drastically suppress or elevate the miR-195-5p levels in TPC1 and IHH-4 cells, respectively (Fig. 2D). miR-195-5p was significantly reduced in the stage III-IV PTC patients (Fig. 2E). Next, Pearson’s correlation analysis revealed that a remarkable opposite relationship between CircRNA NRIP1 and miR-195-5p appeared in PTC tumor tissues (*r*^*2*^ = 0.3703, *P* < 0.0001; Fig. 2F). The above results showed that CircRNA NRIP1 expression in PTC tumor tissues was significantly negatively related with miR-195-5p and overexpressed CircRNA NRIP1 in PTC served as ceRNA adsorption to inhibit the expression of miR-195-5p.

### Knockdown of CircRNA NRIP1 inhibits cell proliferation, invasion and induces apoptosis in PTC cell

**Fig. 3:**
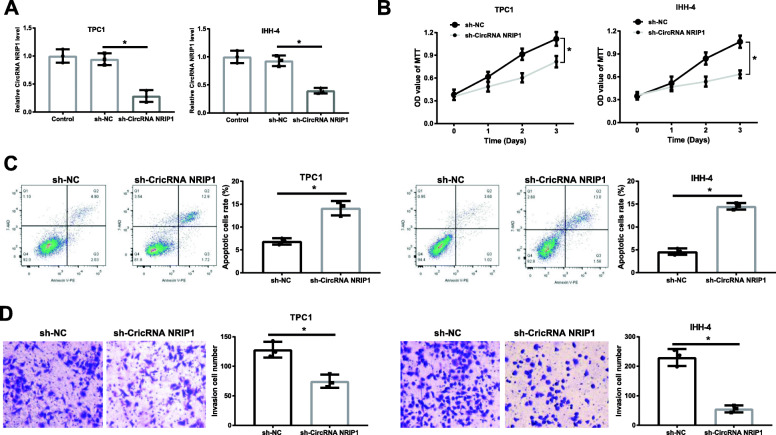
Knockdown of CircRNA NRIP1 inhibited cell proliferation, invasion and induced apoptosis of TPC1 and IHH-4 cells. **A** Interference efficiency measurement in the TPC1 and IHH-4 cells transfected with negative sh-NC or sh-NRIP1 plasmids via qRT-PCR. **B** Proliferation of TPC1 and IHH-4 cells transfected with sh-NRIP1 and sh-NC for indicated times and the proliferative capacity was detected using MTT assays. **C** TPC1 and IHH-4 cells treated with sh-NRIP1 or not were experienced with Annexin V-PE/7-AAD coloration at 48 h and apoptosis was calculated via flow cytometry. **D** The effect of CircRNA NRIP1 knockdown on cell invasion function was performed via transwell assay at 24 h, and the representative pictures of cells migration results were shown. Magnification ×100. *P < 0.05

Considering an abnormal upregulation of CircRNA NRIP1 in PTC, we knocked down CircRNA NRIP1 in TPC1 and IHH-4 cells, and the results reflected that the level of CircRNA NRIP1 was obviously restrained in sh-NRIP1 transfected group compared to sh-NC group (Fig. 3A). MTT assays revealed that CircRNA NRIP1 knockdown resulted in the obvious repression on cell proliferation in TPC1 and IHH-4 cells (Fig. 3B). Flow cytometry analyses revealed that CircRNA NRIP1 depletion contributed to significant acceleration of apoptosis rate in TPC1 and IHH-4 cells (Fig. 3C). The analyses of transwell assays manifested that CircRNA NRIP1 interference strongly inhibited the invasion ability of TPC1 and IHH-4 cells (Fig. 3D). In total, CircRNA NRIP1 silencing evidently enhanced apoptosis and restrained the proliferation and invasion capacities of  TPC1 and IHH-4 cells.

### Effects of CircRNA NRIP1-miR-195-5p axis on PTC cells function

**Fig. 4:**
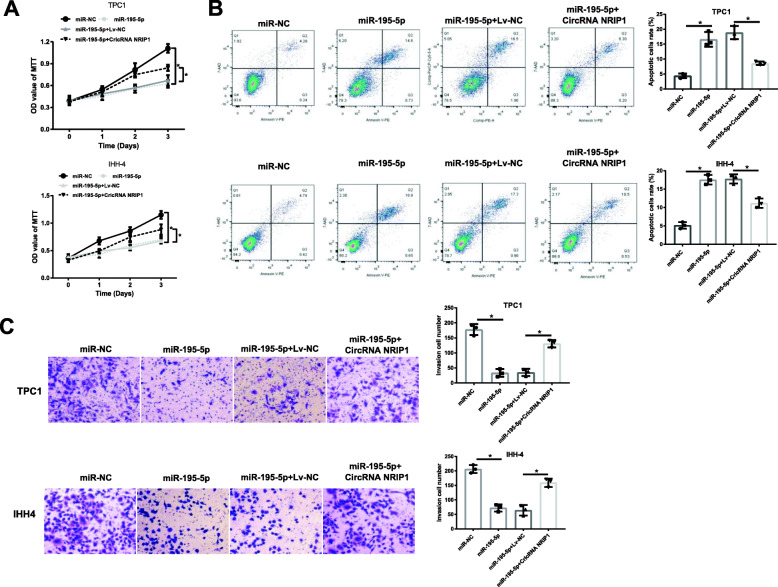
CircRNA NRIP1 enhanced proliferation, invasion and reduced apoptosis of TPC1 and IHH-4 cells by sponging miR-195-5p. **A** Proliferation of TPC1 and IHH-4 cells treated with miR-195-5p or/and CircRNA NRIP1 was measured through MTT assays. **B** The apoptosis of TPC1 and IHH-4 cells with indicated treatment was detected via flow cytometry assay. **C** The invasion capacity of cells was performed by Transwell test at 24 h. Magnification ×100. *P < 0.05

Then, whether CircRNA NRIP1 regulated PTC cells biological behaviors through sponging miR-195-5p was investigated. The elevation of miR-195-5p reduced the proliferative capacities of TPC1 and IHH-4 cells, while the impact of miR-195-5p was mostly abolished following the accompaniment overexpression of CircRNA NRIP1 (Fig. 4A). On the other hand, the enhanced TPC1 and IHH-4 apoptosis by miR-195-5p was effectively weakened by the concomitant enhancement of CircRNA NRIP1 (Fig. 4B). Also, the decreased invasive abilities in miR-195-5p-overexpressed TPC1 and IHH-4 cells were also rescued by CircRNA NRIP1 overexpression (Fig. 4C). Taken together, CircRNA NRIP1 reinforced the harmful functions of PTC cells by sponging miR-195-5p.

### Effects of CircRNA NRIP1-miR-195-5p axis on xenografts growth

**Fig. 5:**
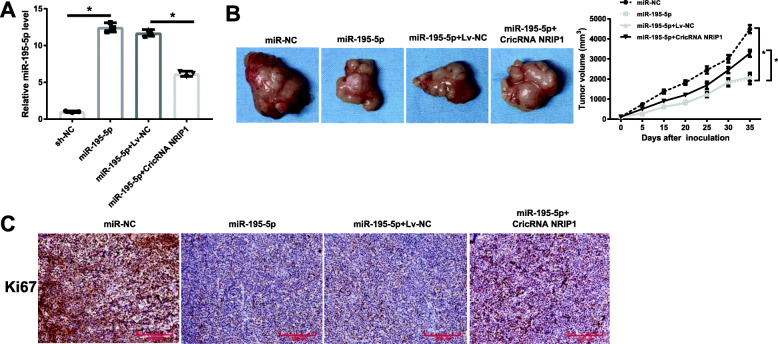
CircRNA NRIP1 promoted the growth of TPC1 xenograft growth by negatively regulating miR-195-5p. **A** The expression levels of CircRNA NRIP1 and miR-195-5p in TPC1 xenograft models were assessed by qRT-PCR. **B** The impact of miR-195-5p, or miR-195-5p and CircRNA NRIP1 in combination on the tumor volumes in xenograft mouse. **C** The proliferation regulator of Ki67 was monitored through immunohistochemistry staining assay after following indicated treatment, scale bar: 200 µM. *P < 0.05

Subsequently, we searched the roles of CircRNA NRIP1 and miR-195-5p in modulating the growth of TPC1 xenografts in mice *in vivo*. As the results demonstrated in Fig. 5A, high level of miR-195-5p was in xenografts models treated with TPC1 infected with miR-195-5p lentivirus, while CircRNA NRIP1 upregulation reduced the expression of miR-195-5p. Strong evidence of the suppressed growth of TPC1 xenografts models was found when there was a rise in miR-195-5p, as testified by the lesser tumor volume in mice inoculated with miR-195-5p in comparison to the control, whereas the effects were rescued by CircRNA NRIP1 overexpression (Fig. 5B). Through immunohistochemistry staining assay, we discovered that miR-195-5p significantly restrained the production of Ki67 compared with control tumors, while the overexpression of CircRNA NRIP1 partially reversed the reduction (Fig. 5C). Our data implicated that miR-195-5p impeded tumor growth *in vivo*, whereas the upregulation of CircRNA NRIP1 effectively renovated the impacts.

### Effects of CircRNA NRIP1-miR-195-5p axis on the P38 MAPK and JAK/STAT pathways in TPC1 and IHH-4 cells and xenografts

**Fig. 6:**
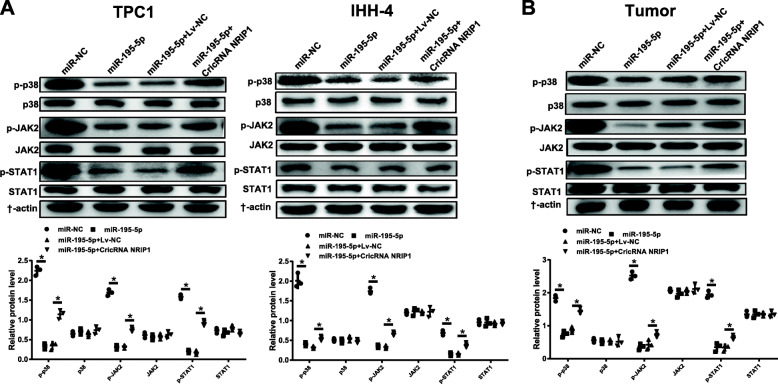
CircRNA NRIP1 activated the P38 MAPK and JAK/STAT pathways by inhibiting miR-195-5p in TPC1 and IHH-4 cells and TPC1 xenograft mouse. **A** and **B** The protein levels of p38 and p-p38, JAK2 and p-JAK2, STAT1 and p-STAT1 were analyzed by western blot in the TPC1 and IHH-4 cells as well as the xenograft mouse. *P < 0.05

In the light of the Western blot results, the protein expression levels of the p-p38, p-JAK2 and p-STAT1 were notably decreased by the miR-195-5p overexpression in both of the TPC1 and IHH-4 cells, while these effects were disturbed following the overexpression of CircRNA NRIP1 (Fig. 6A). In xenografts tissues also exhibited that up-regulation of CircRNA NRIP1 inverted the inhibitive function of miR-195-5p on the P38 MAPK and JAK/STAT pathways (Fig. 6B). These data displayed that CircRNA NRIP1 activated the P38 MAPK and JAK/STAT pathways by inhibiting miR-195-5p in PTC cells and xenograft tumor tissues.

## Discussion

CircRNAs widely exist in eukaryotic organisms. They can participate in the emergence and progression of tumors by regulating gene transcription and alternative splicing, interacting with miRNAs and RNA binding proteins, and affecting cell proliferation, apoptosis, and malignant biological behavior of tumor, which was confirmed a prospective molecular biomarker and therapeutic target for tumor diagnostics with advantages of tissue disease specificity and stability [15–17]. A previous report by Yang et al. [18] has demonstrated that hsa_circ_0039411 is over-expressed in PTC, and miR-1179/ABCA9 and miR-1205/MTA1 are proposed as vital regulatory pathways for the accelerated cell proliferation and invasion capacity and reduced apoptosis of PTC cells. What’s important, Zhang et al. [19] have exhibited that CircRNA NRIP1 sponges miR-149-5p to facilitate gastric cancer (GC) metastasis *in vivo* via inhibiting cell proliferation and migration and activated the AKT/mTOR signaling pathway. Studies have also verified that CircRNA NRIP1 is up-regulated in paclitaxel resistant ovarian cancer tissues and cells and knockdown of CircRNA NRIP1 restrains the drug resistance via sponging miR-211-5p [20]. In the current study, comparing PTC patients and PTC cells with their controls showed that CircRNA NRIP1 was overexpressed. And CircRNA NRIP1 silence suppressed the proliferation, induced apoptosis and restricted the invasion abilities of PTC cells. Furthermore, bioinformatics prediction and luciferase reporter assays and pull down assays verified that CircRNA NRIP1 directly sponged miR-195-5p and inhibited its expression in PTC cells.

miRNAs are a type of small molecule, non-coding regulated RNAs, composed of 19-25 nucleotides, which can regulate gene expression and participate in various biological processes of tumors, and may be an important marker for early tumor diagnosis, treatment and prognosis assessment [21]. For example, studies by Pallante [22], Braun [23] and Peng [24] have reported papillary thyroid carcinoma is correlated with the upregulation of particular miRNAs, for instance miR-146b, miR-199b, miR-221 and miR-222 in comparison to the normal thyroid tissues. These findings reported by Li et al. [25] have confirmed that miR-195 can regulate the levels of cell phase protein D1 and inhibit the viability of cervical cancer cells. Coincidentally, Li et al. [26] have also revealed that there is low-expressed miR-195 in colon cancer tissues and over-expressed miR-195 can inhibit cell viability, migration and invasion through repressing β-catenin, which is a marker for colon cancer diagnosis, and such phenomena have been evidenced in oral squamous cell carcinoma tissues and osteosarcoma [27, 28]. miR-195-5p can reduce the invasion and migration of tumor cells via reducing the production of vascular endothelial cytokines [29]. Recently, Cong et al. have analyzed 499 PTC samples and discovered that miR-195 is down-regulated in the PTC compared to normal thyroid tissues [30]. No less than what is illustrated by Gui et al. [31] that Circ_LDLR sponges miR-195-5p to accelerate papillary thyroid carcinoma metastasis. Intriguingly, our study demonstrated that miR-195-5p played protective action by repressing proliferation and invasion capabilities, facilitating apoptosis of PTC cells and restraining the growth of tumor xenografts, while the functions were all abrogated following CircRNA NRIP1 overexpression in PTC cells and xenografts tissues, which further confirmed that CircRNA NRIP1 could sponge miR-195-5p to accelerate the PTC tumor development.

At present, the cancer-related signaling pathways that are widely involved in thyroid cancer research mainly include RET/PTC-RAS-RAF-MEK/ERK-MAPK, P53-PTEN, RET-β-cateinin, and NF-κB pathways, etc., through which affect the proliferation, apoptosis, invasion and metastasis of tumors [32]. Kim YR et al. [33] have revealed that phospholipase D can synergistically stimulate STAT3 signaling via straightly interference in thyroid oncogenic kinase RET/PTC. Lin H et al. [34] have analyzed the research progress of JAK-STAT signaling pathway and thyroid cancer and concluded that Cdk5 restrains the proliferation of thyroid cancer cells *in vivo* through regulating the STAT3 phosphorylation. The MAPK signaling pathway plays a major role in the adjustment of cell proliferation and the occurrence of human tumors, especially for the occurrence of PTC [35]. In thyroid cancer, mutations in activated BRAF, RAS, and RET-PTC fusion genes can cause the stimulation of the MAPK signaling pathway [36]. Collectively, our western blot results revealed that miR-195-5p upregulation reduced the protein expression levels of p-p38, p-JAK2 and p-STAT1 in PTC cells and tumor xenografts, CircRNA NRIP1 overexpression reversed those effects. The above observations, suggested that CircRNA NRIP1 had the potence to modulate the P38 MAPK and JAK/STAT signaling pathways by sponging miR-195-5p, for which probably serves as a major biomarker in PTC cells by activating downstream proliferation cascade that executes cell activities pathway.

## Conclusions

In conclusion, our findings suggested that CircRNA NRIP1 was up-regulated in PTC tumor tissues and cell lines, and CircRNA NRIP1 silence strained PTC cell proliferation and invasion and accelerated apoptosis. More importantly, CircRNA NRIP1 served as miR-195-5p sponge to modulate the P38 MAPK and JAK/STAT pathways and affected cell functions and growth of xenografts. These findings go far towards the progression of a novel therapy of PTC.

## Supplementary Information

**Additional file 1.**

**Additional file 2.**

## Data Availability

The data used to support the findings of this study are included within the article.

## Abbreviations

CircRNAs: Circular RNAs
NRIP1: Nuclear receptor-interacting protein 1
PTC: Papillary thyroid carcinoma
TC: Thyroid cancer
FTC: Follicular thyroid carcinoma
ATC: Anaplastic thyroid carcinoma
MTC: Medullary thyroid carcinoma
ceRNA: Competitive endogenous RNA
CircRNA NRIP1: CircRNA nuclear receptor-interacting protein 1
FBS: Fetal bovine serum
WT: Wild-type
MUT: Mutant-type
NC: Negative controls
sh-CircRNA NRIP1: Short hairpin RNA against CircRNA NRIP1
sh-NC: Non-specific shRNA
