# Supplementary material for: CircRNA NRIP1 promotes papillary thyroid carcinoma progression by sponging mir-195-5p and modulating the P38 MAPK and JAK/STAT pathways
Source: Diagn Pathol. 2021 Oct 25;16:93. doi: 10.1186/s13000-021-01153-9 (PMC8543861; doi:10.1186/s13000-021-01153-9)
Supplement: Supplementary file 1 — Additional file 1. [file 13000_2021_1153_MOESM1_ESM.docx]

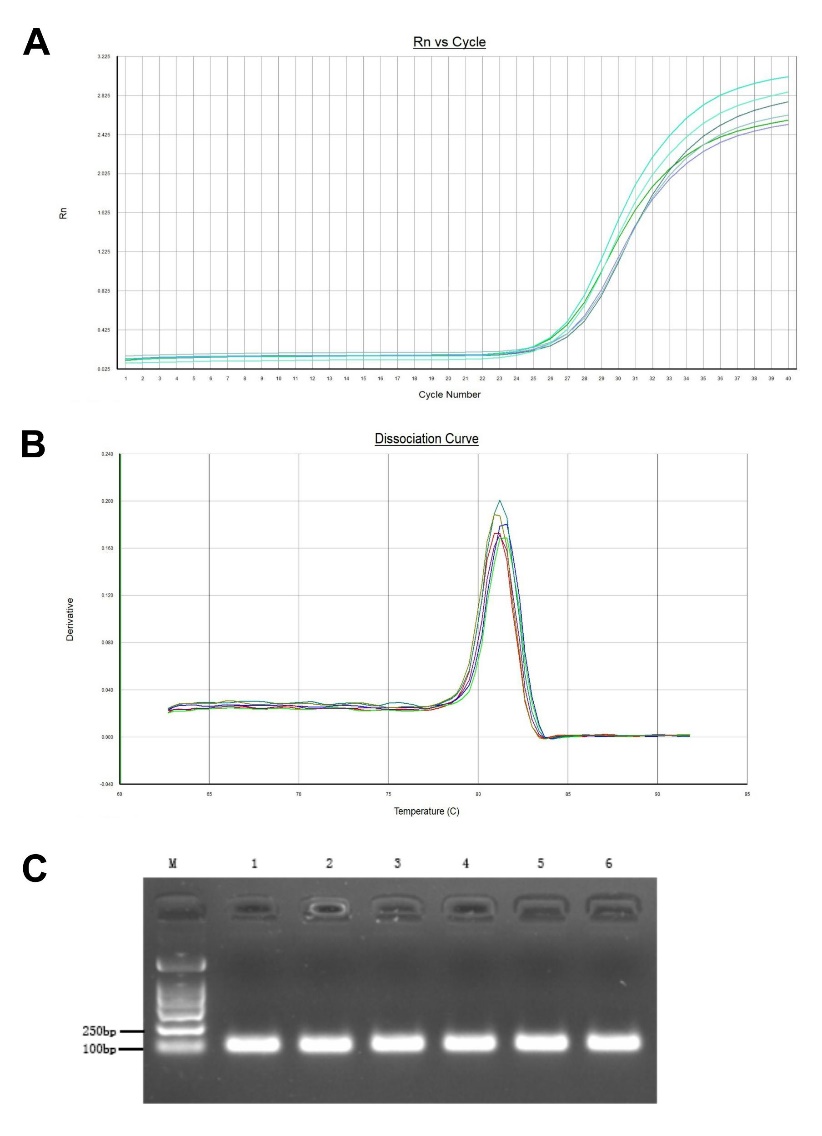


***Figure 1 qRT-PCR results and electrophoresis band for CircRNA NRIP1 before (Figure 1C, lane:1-3) and after treatment (Figure 1C, lane:4-6) with RNase R.***


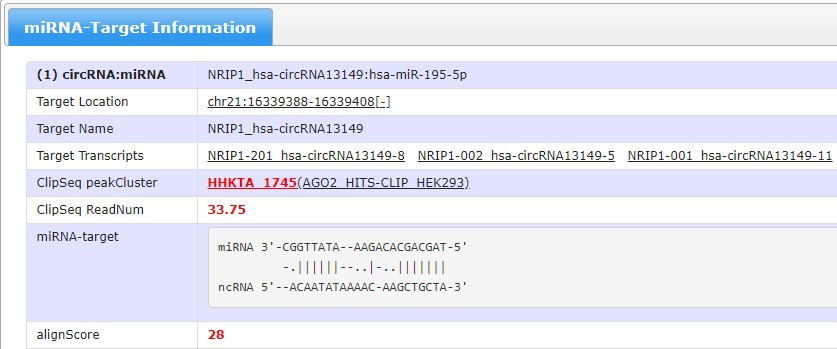
 ***Figure 2 prediction results of the miRNAs viable binding sites in CircRNA NRIP1***


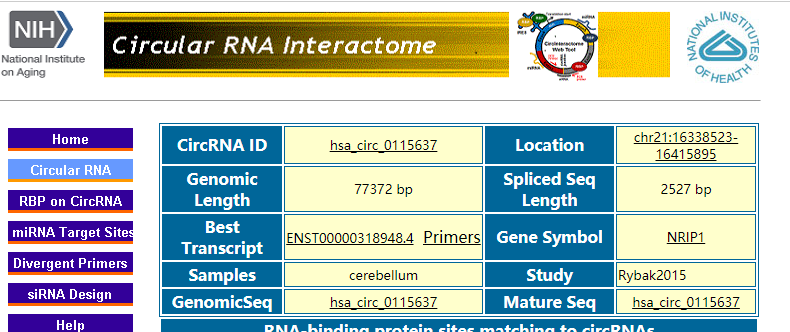


***Figure 3 CircRNA NRIP1 ID is hsa_circ_0115637 on Circ Interactome***


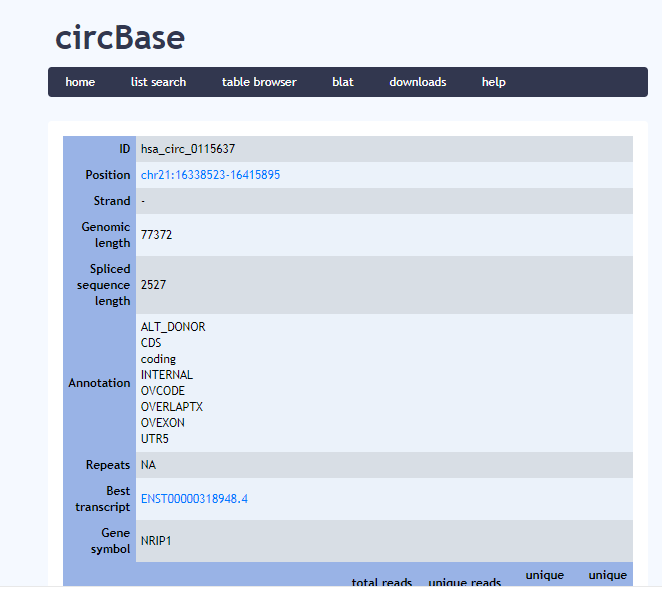


***Figure 4 CircRNA NRIP1 ID is hsa_circ_0115637 on circBase***
