# Supplementary material for: CircRNA NRIP1 promotes papillary thyroid carcinoma progression by sponging mir-195-5p and modulating the P38 MAPK and JAK/STAT pathways
Source: Diagn Pathol. 2021 Oct 25;16:93. doi: 10.1186/s13000-021-01153-9 (PMC8543861; doi:10.1186/s13000-021-01153-9)
Supplement: Supplementary file 2 — Additional file 2. [file 13000_2021_1153_MOESM2_ESM.docx]

| Primers | Sequences (5’-3’) |
| --- | --- |
| has-miR-195-5p-F | 5’-AGCTTCCCTGGCTCTAGCA-3’ |
| has-miR-195-5p-R | 5’-CTGGAGCAGCACAGCCAATA-3’ |
| CircRNA NRIP1-F | 5’- TCAGTGGAAGAGCAGAGACC-3’ |
| CircRNA NRIP1-R | 5’- TCAAGTGTGCATCTTCTGGCT-3’ |
| hsa-U6-F | 5’-CTCGCTTCGGCAGCACATATACT-3’ |
| hsa-U6-R | 5’-ACGCTTCACGAATTTGCGTGTC-3’ |
| GAPDH-F | 5’-AATGGGCAGCCGTTAGGAAA-3’ |
| GAPDH-R | 5’-GCGCCCAATACGACCAAATC-3’ |
| CircRNA NRIP1-WT-F | 5’-GAGCTCGCTAGCCTCGAGCTCGAGCAGAT TGCAAGAAAATGG-3’ |
| CircRNA NRIP1-WT-R | 5’-TTATCATGTCTGCTCGAAGCGGCCGTTCTT TGATATCTACATC-3’ |
| CircRNA NRIP1-MUT-F | 5’-TAAAAATGCAGGTTATAAGAACTCACTG GAAAGAAACAATATAAAACAAGCTG-3’ |
| CircRNA NRIP1-MUT-R | 5’-CAGCTTGTTTTATATTGTTTCTTTCCAGTG AGTTCTTATAACCTGCATTTTTA-3’ |
| PCMV-R | 5’-TCCAAACTCATCAATGTATC-3’ |
| pmiR-GLO-CircRNA NRIP1-F | 5’-GAGCTCGCTAGCCTCGAGCTCGAGCG CGCAGGCAGGGAGAAG-3’ |
| sh-CircRNA NRIP1-F | 5’-CCGGCCGATAGGAAGTGTTTGGATTCAAGACG TCCAAACACTTCCTATCGGTTTTTTG-3’ |
| sh-CircRNA NRIP1-R | 5’-AATTCAAAAAACCGATAGGAAGTGTTTGGACG TCTTGAATCCAAACACTTCCTATCGG-3’ |
| miR-195-5p-F | 5’-GAAGATTCTAGAGCTAGCGAATTCGCAGGTGGTGGA AG-3’ |
| miR-195-5p-R | 5’-CGCAGATCCTTGCGGCCGCGCGGCCGCCTGAAGGTC TCCTTC-3’ |
| CMV-F | 5’-CGCAAATGGGCGGTAGGCGTG-3’ |
| EF1-R | CGCCACCTTCTCTAGGCAC-3’ |
